# Early Detection of Glaucoma and Diabetic Retinopathy in Low-Resource Settings: Barriers and Solutions

**DOI:** 10.3390/jcm15155827

**Published:** 2026-07-25

**Authors:** Esha Gupta, Moe Hein Aung, Eileen Bowden

**Affiliations:** 1 Dell Medical School, The University of Texas at Austin, Austin, TX 78712, USA; 2 Mitchel and Shannon Wong Eye Institute, Dell Medical School, The University of Texas at Austin, Austin, TX 78712, USA; moe.aung@austin.utexas.edu (M.H.A.); eileen.bowden@austin.utexas.edu (E.B.)

**Keywords:** glaucoma, diabetic retinopathy, barriers for ophthalmic care, low-resource setting

## Abstract

Diabetic retinopathy (DR) and glaucoma, globally leading causes of irreversible blindness, can be detected and managed early with timely screening. In low-resource settings, access to ophthalmological examination is limited by a variety of constraints, including scarcity of specialists, equipment costs, geographic distance from care centers, and limited patient awareness. This narrative review examines the principal barriers to early detection of glaucoma and DR and evaluates evidence-based strategies to overcome them, including low-cost portable ophthalmic testing tools, artificial intelligence (AI) analytics, risk-based resource allocation, teleophthalmology, and targeted patient education. In different populations, these strategies have been shown to meaningfully increase access to ophthalmologic screening and follow-up at a significantly lower cost. We describe these approaches, demonstrate previously successful initiatives, and propose various combined approaches adapted to the specific needs of the various low-resource settings.

## 1. Introduction

Diabetic retinopathy (DR) and glaucoma are leading causes of visual impairment and irreversible blindness among adults worldwide [1,2]. Early detection of these conditions is critical for preserving eyesight, as both diseases can progress asymptomatically over years with irreversible structural damage and vision loss often already present at the time of diagnosis [3,4]. Socioeconomic disadvantage and distance from ophthalmology centers have been associated with increased risk of vision loss due to DR and glaucoma, highlighting the need for increased ophthalmological screening solutions for low-resource settings [5,6,7]. This narrative review aims to describe obstacles in the timely detection of DR and glaucoma, and to propose solutions that can be applied to overcome these barriers in low-resource settings.

Barriers to regular ophthalmological screening across low-resource settings may include those faced by providers (such as regional scarcity of eye specialists and high costs of specialized equipment) as well as barriers faced by patients (such as limited education about the conditions, distance from eye care providers, and the costs associated with travel and healthcare services) [8,9,10]. Therefore, inexpensive fundus imaging and portable visual function testing methods that require minimal technical knowledge and skills to operate are potential approaches to enhance early detection of these ocular pathologies. Such resources can be implemented in settings beyond eye-care centers, including primary care clinics, local health fairs, and other healthcare access points. After testing is obtained, diagnosis can be carried out with the use of first-pass artificial intelligence (AI) triaging and asynchronous specialist evaluations, and patient follow-up can be made more accessible through remote counseling.

## 2. Implementing Low-Cost Screening Tools for Fundus Imaging

**Figure 1:**
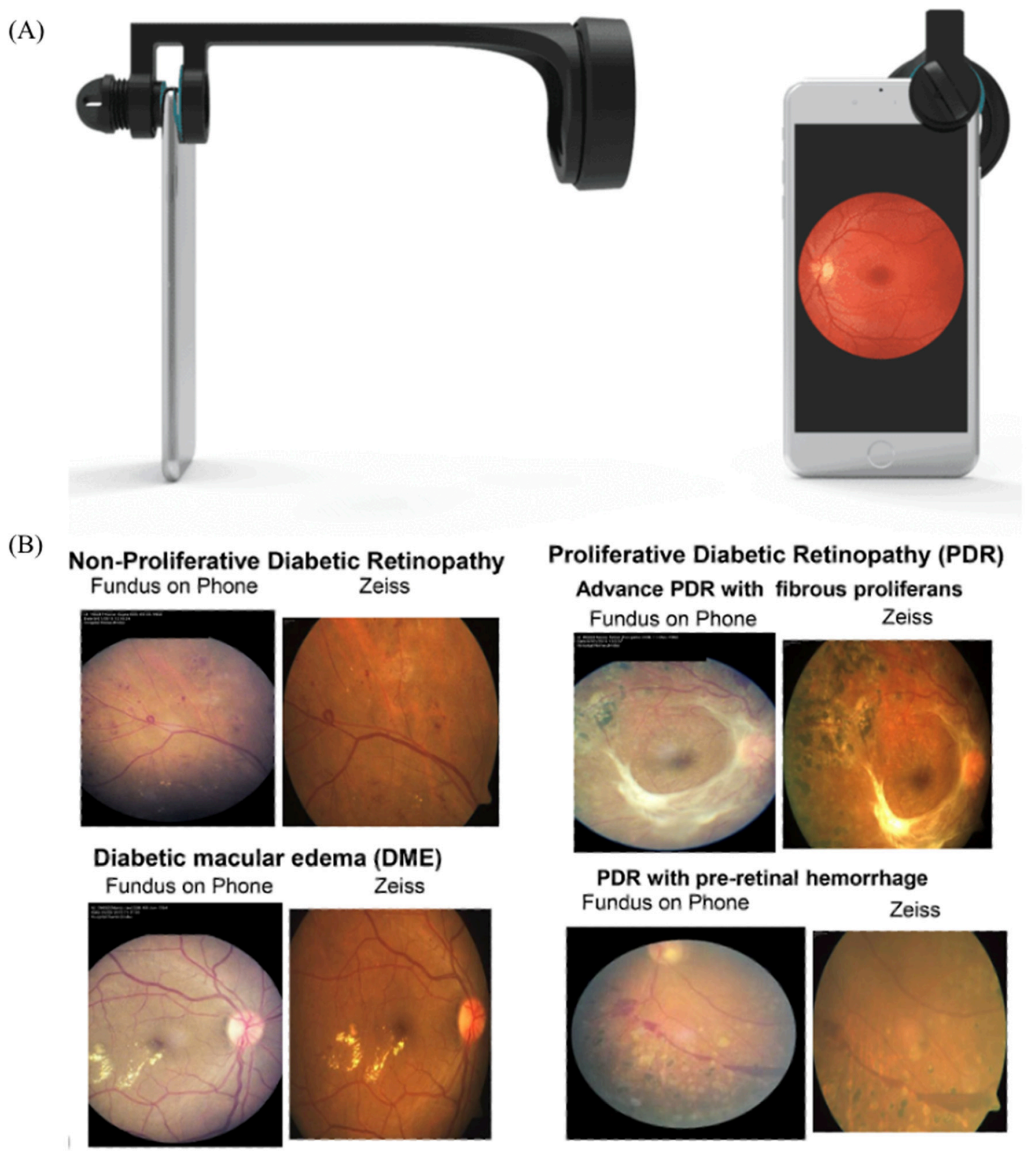
Smartphone-based fundus imaging (SBFI). (**A**) An example setup in which condenser lens is connected to a smartphone via adapter [11]. (**B**) Comparison of retinal images showing signs of diabetic retinopathy captured either by SBFI or Zeiss Fundus camera [14].

Fundus imaging, an essential component of modern ophthalmic care that enables the detection and longitudinal monitoring of diseases such as DR and glaucoma, is often prohibitively expensive, limiting its accessibility in many low-resource and underserved settings. With the advance in the resolution and image quality of smartphone cameras, smartphone-based fundus imaging (SBFI) (based on principles of indirect ophthalmoscopy and consists of a portable condenser lens with an accompanying mobile application) has emerged to provide portable and low-cost examination of potential DR retinal pathologies and glaucomatous optic disc changes (Figure 1A) [11]. There are multiple variations in this setup: the condenser lens may be held in one hand directly in front of the patient’s eye with the smartphone camera positioned in the user’s other hand, or the lens may be attached to the smartphone via an adapter. From its initial publication by Lord et al. (2010), SBFI has become more advanced and widespread [12]. Trials conducted by Adam et al. (2015) found the quality of SBFI images to have comparable assessment value with standard fundus camera pictures [13]. Examples of diabetic retinopathy captured using SBFI compared to Zeiss Fundus camera are shown in Figure 1B [14]. In one example of SBFI tested, Wintergerst et al. (2020) demonstrated a sensitivity of 79% and specificity of 99% to detect DR in diabetic patients seen at outreach clinics across South India [15]. Training healthcare workers to provide SBFI exams is associated with high learnability in relatively short learning periods, highlighting the potential role of primary care physicians and medical technicians in screening a greater number of patients than eye specialists alone [16].

**Figure 2:**
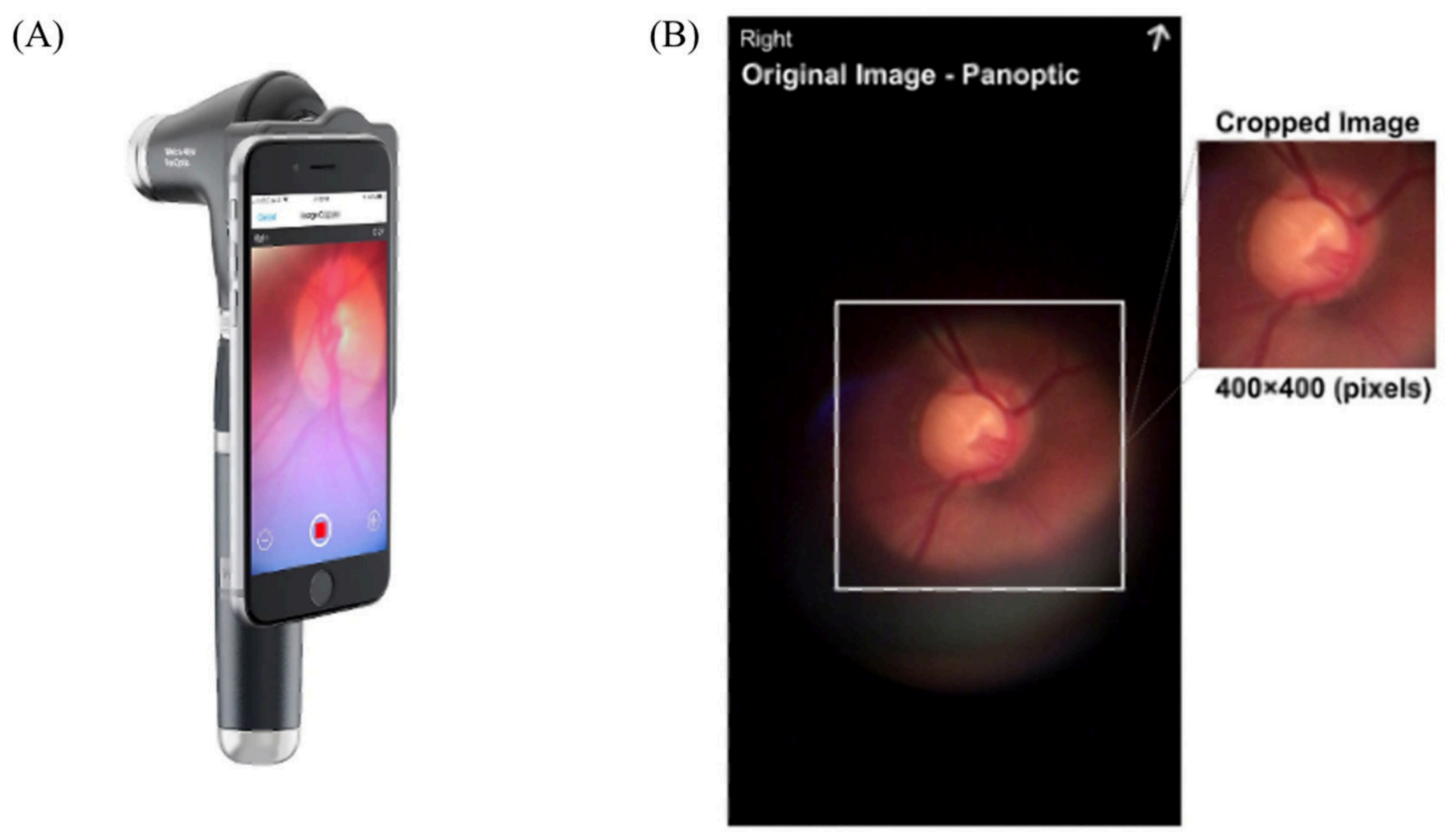
(**A**) iExaminer smartphone adapter for PanOptic Ophthalmoscope [18]. (**B**) Image of glaucomatous optic disc captured with iExaminer [19].

There are a variety of SBFI systems that differ in cost-effectiveness, technical skill to operate, need for pupil dilation, and view of the fundus. For example, imaging via smartphone cameras through a 20D condenser lens may be more useful in the most limited settings, but may require the operator to have a greater degree of technical expertise to capture readable images due to the limited nature of the equipment [17]. For more-resourced centers, a device such as the FDA-cleared iExaminer, which connects one’s phone with the PanOptic ophthalmoscope and its associated mobile application, can capture fundus images with a 25-degree field of view and provide higher-resolution images through undilated pupils (Figure 2A) [18]. An example of a glaucomatous optic disc captured with the iExaminer can be seen in Figure 2B [19]. Giardini et al. (2014) piloted the optical smartphone adapter approach to high-resolution fundoscopy through undilated pupils to measure glaucomatous optic neuropathy and found no difference in glaucomatous optic disc grading when compared to fundus imaging acquired using direct ophthalmoscopy [20]. Similarly, Russo et al. (2016) reported substantial agreement in vertical cup-to-disc ratio grading between slit-lamp biomicroscopy and undilated smartphone ophthalmoscopy for patients with either open-angle glaucoma or ocular hypertension, with exact grading agreement in 72.4% of samples [21]. These findings demonstrate how implementing routine SBFI in low-resource settings can help overcome limitations in accessibility, affordability, and technical knowledge associated with standard retinal fundoscopy.

Although optic nerve photography has been used as a screening tool for glaucoma because of its accessibility and compatibility with teleophthalmology, this approach has limited sensitivity as a stand-alone screening modality, particularly for detecting early functional dysfunctions or subtle structural changes in glaucoma. Comprehensive glaucoma assessment traditionally requires complementary structural and functional testing, including intraocular (IOP) measurement, optical coherence tomography (OCT) analysis, and visual field testing, to improve diagnostic accuracy and disease staging. Though there have been advances in development of more mobile visual field testing devices (will be discussed below), obtaining reliable and accurate IOP measurements in non-ophthalmic clinic setting remain a major challenge. Specifically, IOP readings can vary with measurement technique, corneal thickness, and even daily fluctuations that a single snapshot may not fully capture (a topic of discussion that is outside of the scope of this review). Consequently, IOP monitoring represents a genuine bottleneck in the current system and is particularly vulnerable to inconsistency, limiting how effectively glaucoma can be diagnosed and managed outside of controlled clinical settings.

Nonetheless, for programs selecting an SBFI system, the central choice is between a hand-held condenser lens and a lens fixed to the phone by an adapter, with mydriatic and non-mydriatic options in both categories. Direct comparison of four SBFI devices found that the best-performing device was an adapter that fixed a condenser lens to the phone rather than requiring the operator to hold it [15]. In summary, cost considerations favor the free-hand condenser lens, which requires only a lens and an existing smartphone, whereas ease of use with a quicker learning curve is enhanced with the phone-adapter lens system.

## 3. Implementing Low-Cost Visual Field Testing for Glaucoma

**Figure 3:**
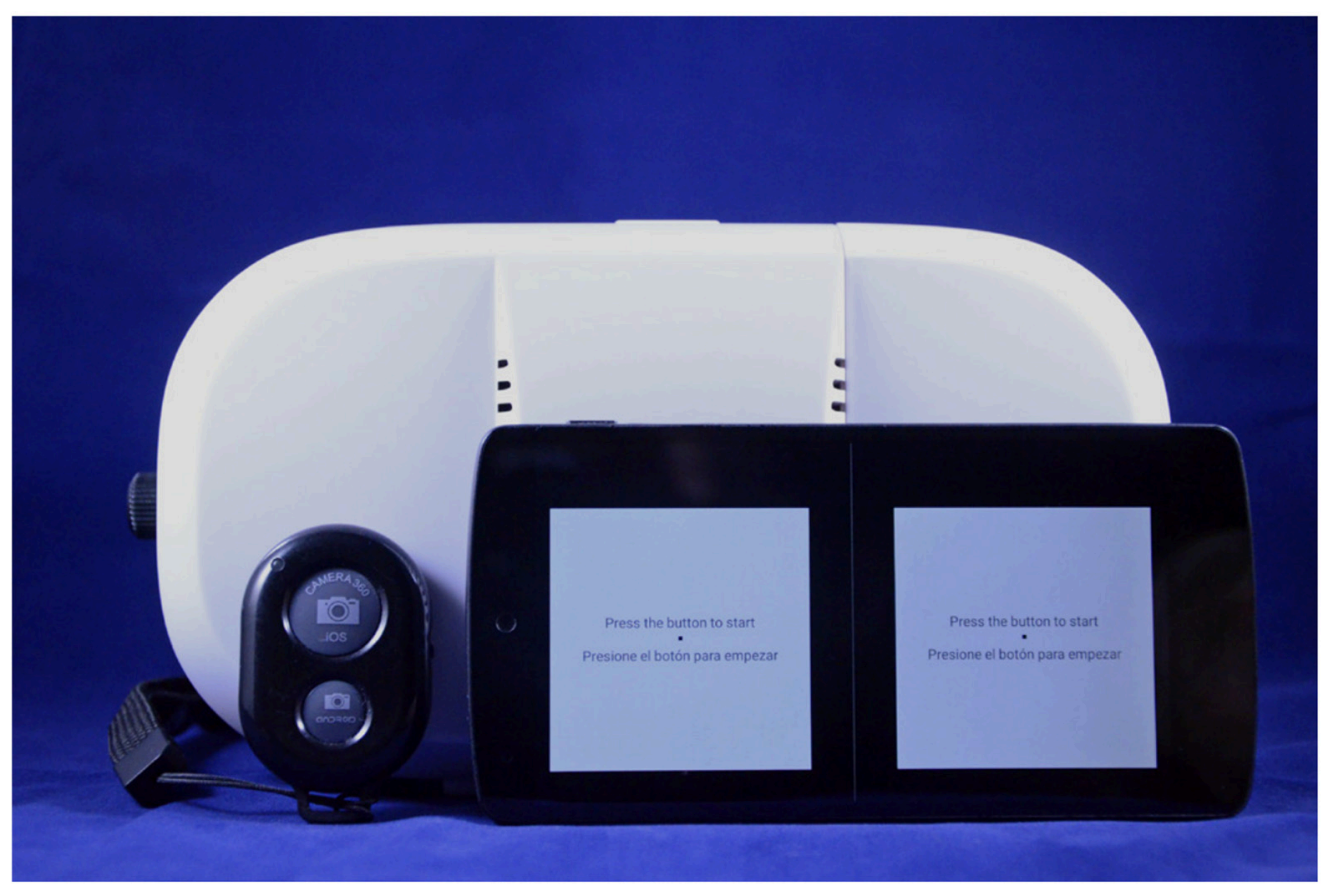
Mobile virtual perimetry model consisting of a smartphone, virtual reality headset, and remote [22].

Monitoring for peripheral visual field changes is essential for glaucoma assessment; however, conventional automated perimetry, such as Zeiss Humphrey Field Analyzer (HFA), is often bulky and costly, underscoring the need for more portable and affordable option. Mobile virtual perimetry (MVP) has been developed as an alternative to traditional table-mounted perimeters, with comparable results. An example developed and tested at the Bascom Palmer Eye Institute Glaucoma Clinic consists of a smartphone-based frequency-doubling technology system with a virtual reality headset and Bluetooth remote (Figure 3), which showed no statistically significant difference compared to the HFA in adult glaucoma patients [22]. Olleyes, a virtual reality-based MVP, allows for visual field testing using a similar headset approach. Olleyes demonstrated 83.8% sensitivity and 94.4% specificity for detecting clinically significant visual field abnormalities, with a positive predictive value of 96% [23]. In glaucoma patients, correlation coefficients for visual field testing between Olleyes and HFA ranged from 0.68 to 0.86 for mean deviation and pattern standard deviation. However, test–retest variability is higher with virtual reality perimetry when compared to HFA; therefore, HFA testing is still preferred and warranted in patients with high clinical suspicion, if available. Tablet and smartphone applications provide additional accessible options. The Visual Fields Easy iPad application demonstrated a sensitivity of 67% and specificity of 77% for identifying abnormal visual fields compared to HFA at a health fair [24]. Additionally, online circular contrast perimetry (OCCP) is a web-based visual field testing application that can be performed on any computer or tablet without additional hardware, making it particularly suitable for low-resource remote monitoring. When administered via a home-based setting, OCCP showed comparable diagnostic accuracy to clinic-based testing, with test–retest intraclass correlation coefficients for mean deviation ranging from 0.90 to 0.93 [25]. Moreover, OCCP was found to maintain acceptable consistency over 6 months of home monitoring [26]. These and other MVP models are portable, more user-friendly, and cost-effective alternatives to traditional visual field testing requiring expensive perimeters in fixed clinical environments, thereby increasing the feasibility of visual field testing for remote and community-based settings with limited resources.

## 4. Improving the Accuracy and Speed of Diagnosis with Artificial Intelligence

Multiple initiatives have studied the implementation of automated AI tools as a first-pass image assessment method for identification of ocular diseases. Deep learning algorithms trained on real patient data are effective in screening for DR and glaucoma, and can be applied to multiple clinical ophthalmological screening modalities such as fundus photos, OCT, and visual fields testing [27,28]. For instance, a primary care clinic serving a low-income metropolitan patient population in Saint Louis, Missouri, USA implemented an AI-automated screening system with non-mydriatic fundus imaging at point-of-care testing. The system was able to detect abnormal results with a sensitivity of 100% (CI 92.3–100%), specificity of 65.7% (CI 57.0–73.7%), and improved adherence to follow-up eye care at the 1-year mark by 36.7% (*p* < 0.0001) [29]. Thus, first-pass AI screening systems to flag and prioritize potentially abnormal imaging results for secondary evaluation by eye specialists can be a very effective tool to improve the efficiency of screening initiatives when the volume of data exceeds the number of specialists to provide timely assessments.

In recent years, Orbis International has provided eye care professionals in low and middle-income countries free access to Cybersight AI, an open-access tool that can detect DR, glaucoma, and macular disease on fundus images [30]. In a limited-resource clinic in Rwanda, the application of Cybersight AI screenings led to accurate referrals for DR and high patient satisfaction [31]. Cost-effective implementation of AI to screen for vision-threatening and referrable DR has also been demonstrated in other low-resource settings in Rwanda, Zambia, and Kenya [32,33,34]. There is ongoing development and comparison of automated tools generated with machine learning for the evaluation of ophthalmic imaging. AI tools developed through the 2018 Retinal Fundus Glaucoma Challenge resulted in the creation of a large public database with reliable references for glaucoma identification and a unified framework for best common practices in the automated evaluation of glaucoma [35]. Moreover, evaluation of fundus images by two of the participating AI models (VRT and SDSAIRC) had resulted in higher diagnostic accuracy when compared to evaluations made by two glaucoma human experts, demonstrating higher sensitivities (VRT: 97.5% and SDSAIRC: 97.6% vs. human experts: 85%) with similar specificity (91.1–91.4%). These findings suggest promising outcomes in the widespread use of AI-interpreted ophthalmic screenings. The increased use of AI in screening, identifying, and monitoring patients is expected to improve the accuracy of remote clinical care of DR and glaucoma.

In addition, several AI systems have been specifically developed and validated for low- and middle-income countries (LMIC) contexts. For example, one non-mydriatic deep learning system trained on images from handheld cameras operated by non-technical field workers across 20 sites in India achieved an Area Under the Receiver Operating Characteristic (AUROC) of 0.99 for referable DR, with 93.9% sensitivity and 96.0% specificity [36]. This study shows that high diagnostic accuracy is possible even with lower-cost imaging and non-specialist operators. Lin et al. (2021) describes a novel Comprehensive AI Retinal Expert model (CARE), which identifies 14 retinal abnormalities and has been evaluated in clinical settings across China, including rural deployment [37]. Lastly, the RETFound-enhanced model, developed using real-world images from community screenings for the purpose of detecting multiple retinal eye diseases, demonstrated improved sensitivity and specificity by >15% over commercial models in community screening, with confirmed benefit in both urban and rural settings via decision curve analysis [38]. Additionally, multiple Markov model analyses have projected that AI-based screening is a particularly cost-effective strategy in low-resource settings. In rural China, AI screening had an incremental cost-effectiveness ratio of $1108/Quality-Adjusted Life Years (QALYs) from the health system perspective, well below cost-effectiveness thresholds. Additionally, the AI-driven screenings outperformed ophthalmologist-based screening, in terms of cost ($180.19 vs. $215.05) and worse outcomes (incremental utility of −0.31 QALYs relative to AI) [39]. These initiatives offer promising AI-assisted eye screening benefits in LMICs. 

Despite high accuracy in controlled studies, AI systems for diagnosing DR and glaucoma share several important limitations, the most fundamental of which is poor generalizability. In a real-world comparative evaluation of seven autonomous DR algorithms over 300,000 images, performance varied substantially between clinical sites, and models trained on data limited to a particular geographic or ethnic group tended to perform worse when applied to a different population in that study, with real-world sensitivities ranging from 51% to 86% and specificities from 60% to 84% [40]. Reported accuracy is also inflated by flawed methodological choices; many studies exclude ungradable images and patients with comorbid eye disease, which does not reflect actual patient populations. In practice, non-mydriatic photography produces variable image quality, and limited diversity in training data and overfitting to research datasets further degrade practical results [41]. For both diseases, unresolved issues of data quality, generalizability, regulation, and workflow integration remain barriers to real-world clinical impact from the use of AI to screen for DR and glaucoma.

## 5. Improving Resource Allocation Using Population Analytics and Risk-Stratification

In addition to examining imaging data for specific ocular disease(s), AI tools can analyze health parameters associated with specific demographic data to identify patients at a higher risk of certain ocular pathologies. Machine learning models applied to population-level data to carry out risk-stratification can meaningfully improve eye screening rates in low-resource settings by identifying patient- and region-specific risk factors, predicting disease burden, and enabling data-driven allocation of limited ophthalmic resources. For instance, a multi-ethnic population study of 10,033 participants used gradient boosting machines to identify significant risk factors, which were diabetes duration (relative influence 22.1%) for DR and socioeconomic factors (mainly educational level, 20%) for glaucoma [42]. The same study also revealed modifiable risk factors, including intraocular pressure, body mass index, alcohol consumption, and hemoglobin A1c (HbA1c), that contributed substantially to glaucoma (~35% cumulative) and DR (20–30%). These findings allow low-resource programs to target interventions toward the most impactful modifiable factors in their specific populations. Similarly, an XGBoost-based classifier, which provides enhanced speed and computation compared to other machine-learning algorithms, used 10 easily obtained non-ocular variables (duration of diabetes, HbA1c, systolic blood pressure, serum triglyceride level, body-mass index, serum creatinine, age, educational level, duration of hypertension, and income level) to achieve an AUROC of 0.816 for detecting referable DR in a rural Chinese population without requiring any retinal imaging [43]. In a separate study, Ravindranath et al. (2024) developed an AI model to prescreen patients with a higher risk for glaucoma based on self-reported health data from surveys sent to patients who had at least two eye-related diagnoses in their electronic health record [44]. Approaches such as these are particularly valuable for pre-screening in settings where fundus cameras are unavailable, allowing limited imaging resources to be directed toward the highest-risk individuals. Financially, targeted screening efforts can reduce the false positive rate and increase the cost-effectiveness of screening programs, potentially increasing their long-term sustainability [45]. Furthermore, the use of AI-based predictive analytics can assess patient risk factors and existing health data to enable longitudinal estimation of disease progression, which can aid eye specialists in providing more personalized care plans earlier on in treatment [46]. The increased use of Al in screening, identifying, and monitoring patients is expected to improve the accuracy of remote clinical care for glaucoma and DR.

## 6. Optimizing Pre-Existing Infrastructure with a Community-Centered Approach

There have been multiple successful models of eye screening initiatives embedded within pre-existing infrastructure across low-resource settings, spanning primary healthcare centers, schools, community health worker networks, and pharmacies. For example, school-based initiatives represent one of the most widely used platforms for embedding eye screening in existing infrastructure in LMICs. A scoping review of 95 studies demonstrated that school-based vision screening is cost-effective and that embedding smartphone screening into existing school health infrastructure can achieve near-complete follow-up [47].

Another particularly successful documented approach embeds retinal screening into routine diabetes care visits at primary healthcare facilities. Examination of 30 studies across India, South Africa, Pakistan, and Bangladesh found that the most effective eye screening models in these LMICs used non-mydriatic fundus photography with AI-assisted or remote specialist grading, integrated directly into primary care diabetes workflow [48]. DR screening embedded within existing diabetes care pathways at primary healthcare facilities was associated with increased follow-up. Of note, training non-physicians (nurses, medical assistants, optometrists) for image acquisition, with specialist grading occurring remotely, was the core feature identified for the success of these models. However, task-shifting of this kind is contingent on a sustained training infrastructure rather than a single training event: structured curricula, competency assessment before independent practice, accessible supervision, and refresher training to offset staff attrition. Therefore, organizations interested in implementing task-shifting will benefit from establishing these resources early on. Umaefulam et al. (2024) detailed multiple validated studies across Asia describing how teachers trained in vision screening perform comparably to primary eye care workers in identifying children with visual disorders, supporting sustainable task-shifting within the school system [49]. In the United States, Federally Qualified Health Centers (FQHCs) serving uninsured and minority populations have demonstrated a similar approach [50]. At one FQHC examined in the study, integrating fundus photography into a comprehensive diabetic care nursing visit raised the annual DR screening rate from 12% to 20%. In rural West Virginia, 33% of patients screened at another FQHC examined in the same study were found to have referral-warranted ocular disease, underscoring the high yield of embedding screening in primary care for high-risk populations.

## 7. Increasing Accessibility and Follow-Up Through Teleophthalmology

Even with effective screening, follow-up attrition remains a persistent challenge in low-resource settings. Telemedicine represents a viable strategy to provide remote eye care for patients residing in such settings. Its use in connecting patients and healthcare providers through virtual platforms has been well-documented in the field of ophthalmology when in-person examination is not feasible, which was especially evident and effective during the COVID-19 pandemic [51,52]. Teleophthalmology describes the use of stereoscopic digital imaging to take ocular images that are transmitted to a specialist for remote assessment and patient counseling [53]. In addition to reducing travel time and costs, teleophthalmology is associated with an average decreased time between referral generation and patient screening (45 ± 22 days vs. 88 ± 47 days; *p* < 0.0001) and reduced appointment duration (78 ± 20 min vs. 115 ± 44 min; *p* < 0.001) compared to standard in-person screening appointments [54,55]. Teleophthalmology has been shown to be a particularly effective screening strategy when the ratio of providers to patients is low, the distance to reach a provider is prohibitive, or the alternative is no patient screening [56]. Furthermore, teleglaucoma screening has been shown to reduce the false-positive referral rate; thereby alleviating systemic burden on both patients and providers and enabling better allocation of resources toward cases requiring specialist intervention [57]. Caffery et al. (2017) examined teleophthalmology models of care in diverse settings around the globe and identified DR and glaucoma as the two most common diseases currently utilizing teleophthalmic screening methods [58]. Both diseases can be detected within the same imaging field of the posterior pole of the eye, highlighting the potential for a combined detection system [58]. For instance, a telemedical DR screening initiative (piloted by the Montefiore Health System in the Bronx, New York, NY, USA) placed nonmydriatic fundus cameras in local primary care sites that serve multi-ethnic, low-income communities and then transmitted the retinal images to an ophthalmologist network for dedicated review [59]. The program was cost-effective, as measured by downstream revenue generated by follow-up visits, and succeeded in identifying patients who may otherwise not have been formally evaluated for ophthalmic conditions, increasing local quality-adjusted life years. These findings highlight the benefit of remote eye care for both patients and providers in low-resource settings, with teleophthalmology increasing access to eye care in underserved or remote areas by enabling timely screening, diagnosis, and management without requiring in-person specialist visits.

A major limitation on the success of teleophthalmology is its accessibility in various settings, which depends heavily on existing technological infrastructure and on patient proficiency with technology. A further constraint is that screening confers no clinical benefit in isolation; remote detection displaces the bottleneck from diagnosis to treatment, and any teleophthalmology program should therefore be coupled to accessible downstream care. Together, these requirements give rise to a central tension: the conditions under which teleophthalmology offers the greatest advantage are frequently the same conditions under which reliable power, connectivity, and referral capacity are least likely to be available. Access-related barriers, rather than the screening technology itself, often determine whether patients ultimately reach care. Teleophthalmology should therefore be regarded as most advantageous under specific structural conditions rather than as a universally applicable solution, reinforcing the need to match the strategy to the resources and referral capacity of each setting.

## 8. Enhancing Patient Education and Engagement Through Outreach Efforts

An additional component to the success of screening efforts in early detection of DR and glaucoma is increasing public awareness about vision-threatening ocular diseases. Health education interventions about DR are significantly associated with an increase in eye screening attendance. For example, educating diabetic patients about DR and the importance of early detection was associated with an increase in motivated behaviors to prevent or address their DR in one qualitative study [60]. A randomized controlled trial conducted with a central Texas diabetic population demonstrated an increased willingness of patients to undergo free ophthalmological screenings when educated about microvascular complications associated with diabetes and essential tests for diabetics via mailed handouts compared to patients who were offered the same free exam but received no education (RR = 2.23; 95% CI = 1.47–3.39; *p* = 0.0001) [61]. Furthermore, digital interventions may be superior for large-scale patient education interventions. For instance, among diabetic patients primarily residing in urban slums of Hyderabad, India, educational videos led to significantly higher diabetic retinopathy screening rates compared to pamphlets (32.7% vs. 11.5%; *p* < 0.05) [62]. Similarly, educational workshops hosted by Philadelphia Wills Eye Hospital at local community sites targeted towards patients at higher risk of developing glaucoma resulted in increased patient awareness of glaucoma, as determined by pre- and post-test score averages (3.86 ± 1.95 vs. 4.97 ± 1.82; *p* < 0.001) and subsequent eye exam appointments [63]. Patient-centered education emphasizing the importance of regular eye exams and adherence to follow-up eye care remains a critical component for the long-term success of screening programs. These examples of proactive counseling increase individual and community understanding that regular screening and timely treatment are essential to preserving vision and maintaining good eye health, in addition to spreading awareness about conditions they may be at risk for.

Additionally, there is strong evidence to support the use of mobile reminder systems to improve screening and follow-up attendance. Among 301 low-income minority patients in Los Angeles, California, automated appointment reminder calls increased DR screening show rates from 46.3% to 59.9% (*p* = 0.036) [64]. Of note, the initiative doubled attendance among African American patients specifically (23.6% → 51.6%; *p* = 0.002), correcting a pre-existing racial disparity. A randomized controlled trial among 233 diabetic patients at five hospitals in low-income Guangdong in rural China demonstrated that automated message reminders containing DR education, sent 1 week and 3 days before appointments, tripled attendance (42.9% vs. 14.0%; RR 3.04; 95% CI 1.73–5.33; *p* < 0.001) while having minimal cost to the healthcare system ($5.40 per person) [65]. In this study, baseline DR knowledge was independently associated with attendance, suggesting that the educational content of the messages contributed to the effect.

## 9. Optimizing a Combined Approach

To review what has been discussed thus far, in Table 1, we summarize the comparative advantages, limitations, cost, and appropriate clinical settings of various strategies to improve early detection of DR and glaucoma in low-resource settings.

Strategies that incorporate different elements of SBFI, MVP, AI screening tools, risk-stratification guiding resource allocation, teleophthalmology or asynchronous care, and patient education campaigns in collaboration with low-resource community health centers can increase medical access and early disease diagnosis. For example, Moinul et al. (2018) demonstrated the success of a combined approach to screening by utilizing remote specialist interpretation of retinal imaging combined with DR education efforts targeted towards high-risk and historically non-compliant DR individuals, resulting in increased follow-up patient adherence (35.6% before the education program vs. 50.5% afterwards; *p* = 0.03) [66]. These findings highlight the importance of integrating educational interventions with screening programs, as improving patient knowledge and awareness can significantly enhance engagement with recommended eye care services. Such strategies are particularly valuable in underserved communities where barriers to healthcare access often contribute to delayed diagnosis and treatment. Furthermore, Rogers et al. (2021) demonstrated the ability of AI retinal evaluation system Pegasus to detect proliferative DR from retinal images with comparable quality to desktop fundoscopic imaging when using a handheld fundus camera to detect and grade referable and proliferative DR (89.4% area under the receiver operating characteristic curve; 95% CI = 88.0–90.7) [67].

The use of portable imaging technologies combined with AI-assisted interpretation may reduce the need for on-site specialists, allowing screening services to reach larger populations while maintaining diagnostic accuracy. In addition, these technologies can streamline referral pathways by rapidly identifying individuals who require further ophthalmologic evaluation. A comprehensive analysis of combined multi-disease screening (DR, age-related macular degeneration, glaucoma, cataracts, and pathological myopia) found that annual AI telemedicine screening was the most cost-effective strategy in both rural and urban areas. In that analysis, retinal images were captured at a local or remote screening site by a trained technician or non-specialist using a fundus camera, and then transmitted digitally to an AI algorithm that then performed automated image grading. The study also found that AI telemedicine showed even more success compared to no screening in rural settings (incremental cost-effectiveness ratio of $244 (95% CI −315 to 1073) [68]. As in these initiatives, selecting which interventions to utilize for different settings should be based on the availability of resources and the characteristic needs of the population being served. A tailored approach that combines technological innovations, community partnerships, and patient-centered education may provide the greatest opportunity to improve screening uptake, facilitate earlier detection of glaucoma and DR, and ultimately reduce vision-related complications among at-risk populations.

**Figure 4:**
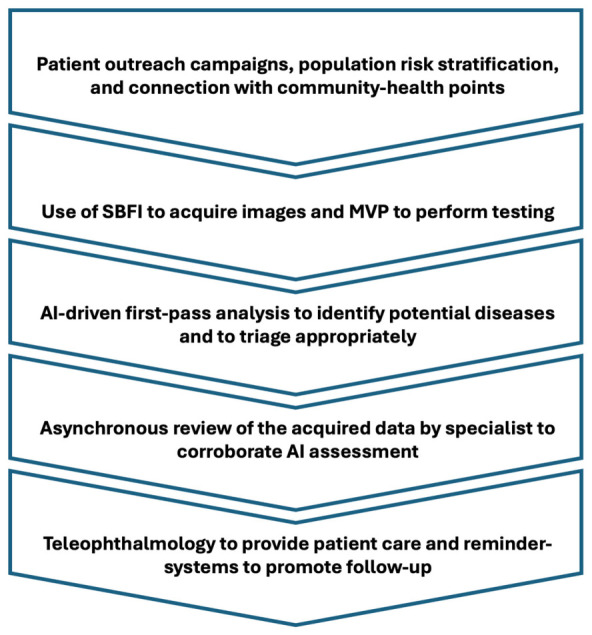
Proposed model for a combined approach to comprehensive ophthalmological screening, analysis, and patient follow-up.

In Figure 4, we will briefly illustrate how a combined screening pathway can be optimized in a low-resource setting by utilizing existing clinical infrastructure. First, a target population is identified through channels such as patient outreach, advertisement, and community risk-stratification. Participating provider(s) can then utilize infrastructure-embedded recruitment strategies (community fair, school event, etc.) to integrate routine diabetes and DR-screening services. In such scenarios, fundal photographs can be captured by local physicians or trained staff and uploaded for asynchronous review with first-pass analysis and triage by AI algorithms. Results that are positive or that remain persistently ungradable are subsequently reviewed asynchronously by an ophthalmologist. Patients with a positive screen are then referred for definitive clinical assessment, with teleophthalmology and structured follow-up incorporated to improve retention and facilitate continued management. With this example of combined approach, we hope to demonstrate how to increase recruitment of at-risk patients while maintaining affordable cost and minimizing additional burden to the existing medical infrastructure.

## 10. Concluding Remarks and Future Directions

Accessibility, affordability, and awareness remain barriers to ophthalmological screening success in low-resource settings. Thoughtful application of the strategies outlined in this article, based on the needs of different low-resource settings, can help overcome barriers in the early detection of DR and glaucoma, an essential step toward advancing equity in vision health. Once implemented, these approaches have the potential to be modified to increase patient follow-up and monitor disease progression when limited accessibility to healthcare facilities remains a barrier for patients. It is important to acknowledge that there is a high degree of variation between different low-resource settings. The initiatives examined in this review article represent a variety of locations, ranging from densely populated yet underserved urban areas in the United States to under-resourced regions globally, highlighting the need for particular attention to differences in sociocultural practices, health literacy, and pre-existing healthcare infrastructure. Therefore, it is important to keep in mind that interventions that are highly effective in one setting may require substantial modification before they can be successfully implemented elsewhere. Additional factors such as transportation access, availability of trained personnel, language barriers, and the accuracy and generalizability of various AI-based programs can all influence program effectiveness. Consequently, local stakeholder involvement and community-based needs assessments are essential for identifying barriers to care and tailoring implementation strategies accordingly. By accounting for these contextual differences, healthcare organizations can improve program adoption, enhance patient participation, and increase the likelihood of achieving long-term improvements in disease detection and management.

It is also important to acknowledge that affordability at the point of implementation does not ensure continuity of these programs. Many published initiatives were started with research or philanthropic funding. However, continuity beyond that initial period generally requires integration into existing financing structures, such as through national health programs, continued grants, or institutional budgets. Funding requirements at the outset, and matching them to what a given setting can realistically sustain, are as decisive for long-term success as the diagnostic performance of the tools themselves. Furthermore, as discussed previously (Section 6), equally pivotal to the financial stability of such programs is establishing and maintaining consistent training programs and standardized workflow protocols to ensure long-term sustainability and effectiveness of these screening systems once implemented.

Though we discussed strategies to improve early screening and detection of DR and glaucoma together in low-resource settings, these are two very distinct ophthalmic diseases. Unlike DR, there is currently no universally accepted population-based screening strategy for glaucoma, reflecting the disease’s complex pathophysiology and the absence of a single highly sensitive and specific screening test. While structural assessment via fundus photography and functional testing through visual fields provides critical complementary data, certain treatment decisions, such as adjusting topical therapy to escalate toward surgical intervention, remain heavily dependent on reliable IOP trends. Home tonometry or measurements taken outside of the controlled clinical environment introduce operator-dependent variability. Therefore, detecting early glaucoma and establishing a definitive diagnosis remotely remains imperative, underscoring the need for multimodal screening approaches and careful integration of emerging technologies into clinical care.

Lastly, despite significant progress has been made in expanding access to eye screening for DR and glaucoma through the strategies discussed in this review article, important gaps remain in understanding how these interventions perform across different populations and healthcare environments. Additionally, long-term outcomes related to patient adherence, cost-effectiveness, scalability, and integration, such as those carried out in the various initiatives examined in this article, remain incompletely understood in different settings. Further efforts should continue characterizing and optimizing novel models of ophthalmological screening in different low-resource settings with diverse demographics and needs. Long-term cost-effectiveness analyses should extend beyond initial implementation costs to evaluate program sustainability, patient adherence, program utilization, and visual outcomes over time. In parallel, studies should assess the financial and operational sustainability of screening programs by examining local infrastructure needs, maintenance costs, and integration with existing referral networks. As artificial intelligence becomes increasingly incorporated into ophthalmic screening, further validation across diverse patient populations is essential to ensure equitable performance and minimize algorithmic bias. Future research should prioritize prospective, multicenter validation studies to evaluate the real-world diagnostic performance, clinical utility, and generalizability of these emerging DR and glaucoma screening technologies across diverse healthcare settings.

## Figures and Tables

**Table 1:** Comparative summary of strategies to enhance the early detection of and outcomes related to DR and glaucoma in low-resource settings.

Strategy	Advantages	Limitations	Relative Cost	Appropriate Clinical Setting
Smartphone-based fundus imaging	Portable; detects retinal pathologies and optic disc changes with moderate image quality; high learnability; non-mydriatic options	Performance, and ease-of-use varies by system	Low to moderate cost depending on resolution	Primary care clinics, health fairs, outreach clinics, and other community access points
Mobile virtual perimetry	Portable, user-friendly; results comparable to HFA; some options need no additional hardware and support remote monitoring	Higher test–retest variability than HFA, so HFA remains preferred for patients with high clinical suspicion	Low to moderate	Remote and community-based settings, home monitoring, health fairs
AI-assisted screening	Improves efficiency when volume exceeds specialist capacity; high sensitivity and specificity in validated studies	Varied generalizability across sites and populations; real-world accuracy lower than in controlled studies; regulatory and workflow-integration barriers remain	Low marginal cost per use once deployed	Point-of-care primary care, high-volume screening centers
AI-assisted risk stratification of population data	Allows value-based direction of limited screening resources; can pre-screen for pathology	Depends on quality of available population and health data; identifies risk-factors but does not confirm a diagnosis	Low	Pre-screening; program planning and resource allocation
Teleophthalmology	Reduces travel time and cost; shortens referral to screening interval; especially valuable where provider-to-patient ratios are low or distances prohibitive	Follow-up attrition persists; requires patients to have access to technology and connectivity	Moderate depending on existing staff and infrastructure	Remote or otherwise less accessible areas
Community-based screening centers	Leverages existing health infrastructure; improved attendance	Requires local coordination and reliable infrastructure; may still depend on remote specialist grading	Low to moderate	Schools, primary healthcare centers, community health centers
Patient education	Increases patient intake and follow-up; readily tailored to local populations	Requires patient literacy and understanding; benefit varies across populations	Low	Adjunct across all settings
Reminder systems	Associated with substantially increased appointment attendance; scalable and easily integrated into communication systems	Requires mobile access	Very low	Adjunct across all settings; appointment scheduling, post-visit follow-up

## Data Availability

No new data were created.

## Abbreviations

The following abbreviations are used in this manuscript:

AI	Artificial Intelligence
DR	Diabetic Retinopathy
HFA	Humphrey Field Analyzer
HbA1c	Hemoglobin A1c
MVP	Mobile Virtual Perimetry
OCCP	Online Circular Contrast Perimetry
OCT	Optical Coherence Tomography
IOP	Intraocular Pressure
FQHC	Federally Qualified Health Centers
AUROC	Area Under the Receiver Operating Characteristic
LMIC	Low- and Middle-Income Countries
QALY	Quality-Adjusted Life Years
